# Demographics, reproduction, growth, and abundance of Jollyville Plateau salamanders (*Eurycea tonkawae*)

**DOI:** 10.1002/ece3.3056

**Published:** 2017-05-31

**Authors:** Nathan F. Bendik

**Affiliations:** ^1^ Watershed Protection DepartmentCity of AustinAustinTXUSA

**Keywords:** natural history, Plethodontidae, reproductive phenology, von Bertalanffy

## Abstract

Insights into the ecology and natural history of the neotenic salamander, *Eurycea tonkawae,* are provided from eight years of capture‐recapture data from 10,041 captures of 7,315 individuals at 16 sites. *Eurycea tonkawae* exhibits seasonal reproduction, with peak gravidity occurring in the fall and winter. Size frequency data indicated recruitment occurred in the spring and summer. Open‐population capture‐recapture models revealed a similar seasonal pattern at two of three sites, while recruitment was dependent on flow at the third site. Females can reach sexual maturity within one year, and oviposition likely takes place below ground. The asymptotic body length of 1,290 individuals was estimated as 31.73 mm (at ca. two years of age), although there was substantial heterogeneity among growth trajectories. Longevity was approximately eight years, and the median age for a recaptured adult was 2.3 years. Abundance estimated from closed‐population and robust‐design capture‐recapture models varied widely within and among sites (range 41–834), although, surprisingly, dramatic changes in abundance were not observed following prolonged dry periods. Seasonal migration patterns of second‐year and older adults may help explain lower ratios of large individuals and higher temporary emigration during the latter half of the year, but further study is required. Low numbers of captures and recaptures precluded the use of open‐population models to estimate demographic parameters at several sites; therefore, closed‐population (or robust‐design) methods are generally recommended. Based on observations of their life history and population demographics, *E. tonkawae* seems well adapted to conditions where spring flow is variable and surface habitat periodically goes dry.

## 1 INTRODUCTION

Natural history data are important for informing conservation, management, and policy for imperiled species, although these data are difficult to collect, require considerable time and effort, and are infrequently the focus of scientific inquiry (Bury, 2006; Dayton, 2003; Greene, 2005; Greene & Losos, 1988). Within the Edwards Plateau of central Texas, thirteen salamander species (genus *Eurycea*, clade *Paedomolge*, sensu Hillis, Chamberlain, Wilcox, & Chippindale, 2001) inhabit a range of karst‐associated aquatic habitats, from hillside seeps to large springs and expansive stream networks both above and below ground (Chippindale, Price, Wiens, & Hillis, 2000; Sweet, 1978; Sweet, 1982). Seven species in this group are federally listed as threatened or endangered, and have small distributions within and around urbanized and rapidly developing areas (Chippindale & Price, 2005; US Fish and Wildlife Service 2013, 2014a). However, detailed information on reproductive phenology, population demographics, and life history for this group is limited (but see Bruce, 1976; Tupa & Davis, 1976; Pierce, McEntire, & Wall, 2014).

**Figure 1:**
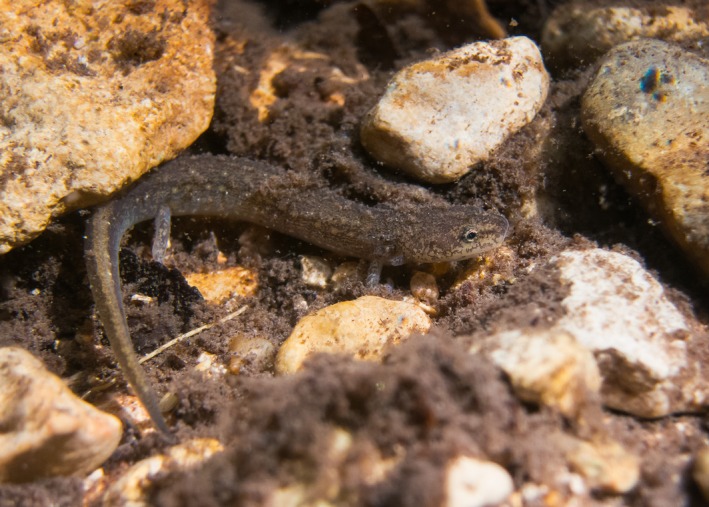
Adult *Eurycea tonkawae* in Bull Creek, Travis County, Texas

Jollyville Plateau salamanders (*Eurycea tonkawae*; Figure 1) are restricted to northwestern Travis and southern Williamson counties, Austin, Texas (Chippindale et al., 2000), and are federally listed as threatened (US Fish and Wildlife Service 2013). The first published account on the ecology of *E. tonkawae* was by Bowles, Sanders, and Hansen (2006), who examined monthly count data from a two‐year period to assess relationships between relative abundance, habitat characteristics, and urbanization. Recent studies have extended this work, including estimation of demographic parameters (O'Donnell & Gluesenkamp, 2009), relative abundance in the context of land‐use patterns (Bendik, Sissel, Fields, O'Donnell, & Sanders, 2014), and movement and occupancy within headwater streams (Bendik, McEntire, & Sissel, 2016).

In this study, I examined the natural history of the *E. tonkawae* by summarizing capture‐recapture and body size data from an 8‐year period from 16 sites across its geographic range. I analyzed body size frequency distributions to characterize demographic and reproductive patterns and compared these results to other central Texas *Eurycea*. From the capture‐recapture data, I modeled individual growth and generated estimates of age to determine the timing of life history events. I also estimated demographic parameters using open‐ and closed‐population capture‐recapture models. Collectively, this work provides new and important insights into the ecology and natural history of this microendemic species.

## 2 METHODS

### 2.1 Study sites and data collection

**Figure 2:**
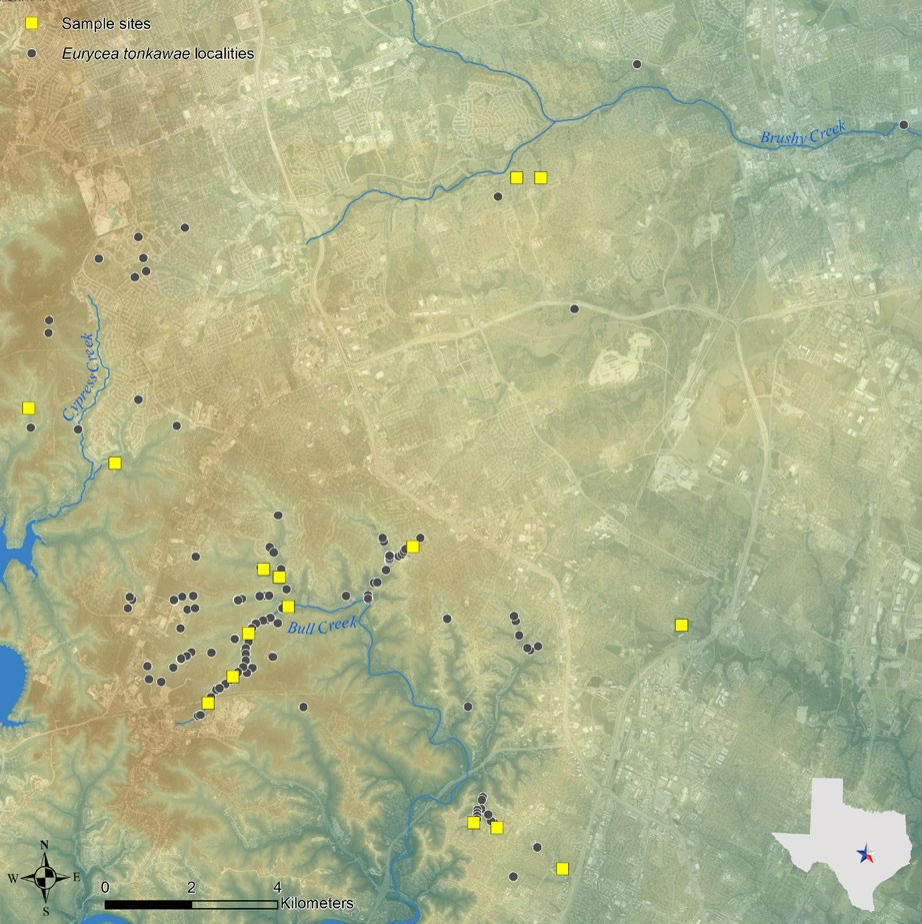
*Eurycea tonkawae* localities and study sites

Sites were located within and around the Jollyville Plateau physiographic region of central Texas and were distributed across the range of *E. tonkawae* (Figure 2). All of the study sites and survey areas were adopted from prior studies (Bendik et al., 2014, Bowles et al., 2006; City of Austin 2001; O'Donnell & Gluesenkamp, 2009). Fixed survey areas were delineated within and around springs, although survey areas varied substantially among sites (Table 1) due to differences in spring and stream flow (and thus, available habitat) and judgments made by the original investigators. These sites varied in quality and were distributed throughout the range of the species, from heavily urbanized areas to large preserves (Bendik et al., 2014).

**Table 1:** Study site information for surveys of *Eurycea tonkawae* performed from 2008 to 2015

Site	Type	Survey area (m^2^)	*n* Surveys^a^ (total)	*n* Surveys^b^ (CMR analyses)	*n* Captures	*n* Individuals
Avery Deer	Headwater spring	7.5	12	–	131	123
Balcones	Main channel spring	4.9	13	–	3	3
Barrow	Main channel spring	33.3	12	–	143	106
Franklin	Main channel spring	68.3	15	13	1,951	1,307
Hill Marsh	Headwater spring	19.7	14	14	1,355	777
Lanier	Main channel spring	64.5	43	9*	2,584	1,840
Lower Ribelin	Main channel spring	52.4	15	4*	820	729
MacDonald Well	Main channel spring	61.9	1	–	43	43
Spicewood	Headwater spring	40.0	10	–	9	8
Stillhouse	Headwater spring	28.9	9	–	45	45
Tanglewood	Headwater spring	23.4	8	–	12	12
Trib 5	Main channel spring	52.7	14	–	122	92
Trib 6	Main channel spring	109.5	16	–	601	453
Troll	Headwater spring	17.6	15	14	311	230
Upper Ribelin	Main channel spring	43.8	10	–	691	551
Wheless	Headwater spring	90.5	16	5*	1,220	996
Totals			186		10,041	7,315

^a^Total number of surveys performed, including repeated sampling for closed‐population surveys.

^b^Number of surveys used in capture‐recapture modeling; *indicates number of primary periods for closed‐population surveys.

Surveys performed during this study represent a continuation of several population monitoring efforts of *E. tonkawae* at 16 sites which were subsequently extended to obtain multiyear capture‐recapture data. The sampling followed either a closed‐population capture‐recapture protocol following O'Donnell and Gluesenkamp (2009) or an open‐population capture‐recapture protocol at sites where sampling initially consisted of population counts (Bendik et al., 2014, Bowles et al., 2006; City of Austin 2001). The closed‐population method (Otis, Burnham, White, & Anderson, 1978) entailed successive sampling (three days in a row) during a short enough period (the “primary” period) where demographic closure (no births, deaths, or migration) of the population was assumed. Sampling under the open‐population method (Nichols, 2016) occurred over longer time intervals (generally > three months), during which the population was open to demographic change. When multiple closed‐population samples are combined to include intervals where the population is open to demographic change, this is referred to as the robust‐design (Pollock, 1982).

From 2008 to 2012, surveys were performed at three sites within the Balcones Canyonlands Preserve using closed‐population capture‐recapture sampling (Table 1). Although quarterly sampling events were desired, severe drought hampered the initial attempt to collect data at evenly spaced intervals. Thus, sampling for these sites was generally opportunistic during the study period. From 2012 to 2015, open‐population surveys were performed at 15 sites (including two sites from the closed‐population sampling). This includes surveys that were either opportunistic, performed as part of another study, or was part of a prescribed monitoring plan. Opportunistic sampling includes single surveys (e.g., MacDonald Well; Table 1) or surveys to collect additional demographic and individual information on previously marked animals. Data are also included from other studies where individual salamanders were identified. This includes a four‐month open‐population capture‐recapture study of movement (Bendik et al., 2016) and a study of hormone levels (Gabor et al., 2016). Finally, nine sites were regularly sampled once per quarter during February/March (winter), May/June (spring), August/September (summer), and November/December (fall). However, springs periodically ceased flowing during our study which resulted in ragged survey intervals and incomplete sampling for some years and sites. Please refer to Table 1 for additional site‐specific information.

Salamanders were found by exhaustively searching available cover objects including gravel, cobble, leaves, and woody debris. Surveyors also searched through vegetation and disturbed fine sediments, using small aquarium nets to capture salamanders. Salamanders were flushed from dense leaf litter or vegetation into large pool nets. Individuals were placed in mesh containers in the water before and after processing.

Individual marking was initially accomplished using visible implant elastomers (VIEs; Northwest Marine Technology Inc., Shaw Island, WA, USA) from 2008 to 2011. Salamanders ≥ ca. 16 mm snout–vent length (SVL) were anaesthetized in a solution of 0.25 g tricaine methanesulfonate (MS‐222)/L of naturally buffered spring water and then marked using VIE tags. Sterile 28‐gauge syringes were used to inject small amounts (2–20 μl) of elastomer just underneath the skin to form a bead. Each salamander was given three to four unique VIE tags using a combination of seven different colors in five locations on the body. All captured salamanders were photographed on a standardized grid background in a water‐filled tray using a DSLR and wireless flash. Marking with VIEs was replaced from 2012 onward with photographic identification using the software Wild‐ID (Bolger, Morrison, Vance, Lee, & Farid, 2012) due to the decreased cost and invasiveness, as well as increased speed and accuracy of photographic identification for this species (Bendik, Morrison, Gluesenkamp, Sanders, & O'Donnell, 2013). This savings in time and cost facilitated an expansion of capture‐recapture monitoring to include more sites.

Salamanders were assessed for gravidity by visually checking for yolked oocytes (the candling method; Gillette & Peterson, 2001). Gravid females were considered sexually mature. Otherwise, individuals were not sexed due to the difficulty of candling for determining presence or absence of testes. Body length (BL) was quantified as the mid‐vertebral distance from the tip of the snout to the posterior insertion of the hindlimbs. Total length (TL) was quantified as the distance from the tip of the snout to the tip of the tail. BL and TL were measured to the nearest 0.1 mm using ImageJ software (Rasband, 1997). To allow for comparisons across different studies, BL measurements were converted to SVL with the following linear regression formula: SVL = 1.032 × BL + 0.896 (*n *=* *14, *r* ^2^ = .99). I summarized body length and gravidity data to assess patterns in population demographics and reproduction.

### 2.2 Capture‐recapture models

I used robust‐design capture‐recapture models (Kendall, Nichols, & Hines, 1997) to estimate abundance (*N*), apparent survival (*φ*), conditional capture and recapture probabilities (p* and *c*, respectively), and temporary emigration (*γ*) at Lanier Spring from 2008 to 2012. For each primary period, I first determined the best model structure for the conditional capture and recapture probabilities from closed‐population models. I included the best structure for that period in the robust‐design model based on Akaike's information criterion corrected for small sample size (AICc; Burnham & Anderson, 2002). I tested models with (1) time‐varying p* and no behavioral effect, (2) a behavioral effect but no time variation (p* and *c* constant within the 3‐day period), and (3) no behavioral or time effect (p* = *c*). I included a behavioral effect because salamanders that were captured and manipulated for marking and/or photography may exhibit a negative response to capture, thus affecting their recapture probability. For the full robust‐design models, apparent survival and temporary migration varied by time. I did not assess models with constant survival and temporary migration, or with covariates on these parameters, because of the high variability of time intervals between surveys. I compared models where temporary migration was either independent (random emigration; *γ*′ = *γ″*) or dependent (Markovian emigration; *γ*′, *γ″*) upon the previous state (Kendall et al., 1997). The final temporary migration (*γ*_*k*_ and *γ*_*k*−*1*_) and apparent survival parameters (*φ*_*k*_) are confounded (Kendall et al., 1997) and were therefore excluded from the results. Survival was assumed to be the same for animals regardless of their probability of capture. Data from Lower Ribelin and Wheless were excluded from the robust‐design analysis due to small sample sizes. Instead, for these populations, I used closed‐population models (as above) to generate estimates of abundance and conditional capture probability for each period.

Jolly–Seber (JS) models allow estimation of apparent survival and effective capture probability (*p* ^*0*^) in addition to population entry probabilities (Pollock, Nichols, Brownie, & Hines, 1990). I used the Link‐Barker JS formulation (Link & Barker, 2005), which parameterizes population entry as per capita recruitment (*f*). This parameter quantifies entrants to the population, either from immigration or births. Preliminary analyses revealed that models with full time variation of parameters were unsupported by the data, so constraints were placed on *φ*,* f*, and *p* ^*0*^ to vary either as a function of season (corresponding to the quarterly interval for spring, summer, fall, or winter), stream flow (total discharge in ft^3^/s), or as constant among all periods. Flow was measured using a Marsh‐McBirney FLO‐MATE model 2000 (Hach Company, Loveland, CO, USA) at the downstream end of each site. I replaced a single missing value for flow from each site with the site mean. Steam flow affects important physical and chemical attributes of the salamander's environment, including water quality, quantity, and depth, each of which may influence demographic rates. For example, high flows during wet periods may scour habitat, reducing the available cover for salamanders and their prey, while very low flows may induce migration away from the surface to avoid desiccation (e.g., Bendik & Gluesenkamp, 2013). The ecology of these oligotrophic headwater streams (Mabe, 2007) is also influenced by seasonal variability in water temperature and nutrient input (e.g., via leaf fall).

I used JS models to examine demographic patterns at three sites (Franklin, Hill Marsh, and Troll). Low rates of recapture and low overall abundance (in particular at several highly urbanized sites; Table 1, Bendik et al., 2014) resulting in small sample sizes precluded application of open‐population capture‐recapture models for other sites. Sites Franklin, Hill Marsh, and Troll were ideal for testing hypotheses about the influence of seasonality and discharge on demographic parameters because spring discharge was uninterrupted during the 3‐year sampling period and the sampling seasons were generally of equal length. The Franklin site is the largest of the three (both in terms of discharge and sample area), and occurs within the main channel of Bull Creek, receiving both spring water from nearby outlets (Pit Spring) and periodic stream flow from a large, mostly undeveloped catchment. Troll Spring sits at the headwaters of a tributary of Bull Creek (Tributary 3). It receives flashy discharge from a highly developed upland catchment, but only during rain events. Hill Marsh Spring occurs within a golf course in a moderately developed area along Brushy Creek, but does not receive any upland discharge. The last period for Troll and Hill Marsh encompassed 3 quarters and was therefore modeled independently of season‐specific effects. The population for each site was defined as salamanders large enough to be “marked” (ca. >16 mm SVL) that used the study area.

Jolly–Seber models require several assumptions, which if not met could result in biased parameter estimates (Pollock et al., 1990). These assumptions include the following: (1) marked animals are recognized accurately and marks are not lost, (2) sampling is instantaneous, (3) marked animals have the same capture probability as unmarked animals, (4) every animal in the population has the same probability of capture in a given sampling period, (5) every marked animal has the same probability of survival between sampling periods, and (6) emigration is permanent. Based on the study design and the use of photographs instead of physical marks, assumptions 1 through 3 are unlikely to be violated. There is some evidence of size‐biased capture and survival heterogeneity in *E. tonkawae* (assumptions 4 and 5) from a previous study (Bendik et al., 2016), although that study included very small juveniles (<16 mm SVL). Small juveniles can be particularly difficult to capture and identify reliably, and have lower survival rates compared to larger animals (Bendik et al., 2016); therefore, I excluded those individuals from the capture‐recapture analysis. The last assumption is the most difficult to meet given prior evidence of movement and temporary emigration at some sites (Bendik et al., 2016; O'Donnell & Gluesenkamp, 2009). If temporary emigration is random, parameters may be unbiased, while Markovian movement can result in biased parameter estimates (Kendall et al., 1997; Nichols, 2016). Given these points, it is important to consider the potential consequences of violating assumption 6 and interpret the JS estimates with caution.

I used program MARK (v8.1; White, 2015) to fit all capture‐recapture models and estimate parameter means and 95% confidence intervals (CI). I checked parameters estimated at the boundary (0 or 1) using the data cloning option in MARK to verify they were actually being estimated (at 0 or 1). I used model‐averaged estimates if the top model had an AICc weight <0.9.

### 2.3 Growth models

I assessed growth of *E. tonkawae* using a hierarchical von Bertalanffy (VB) model that accounts for individual heterogeneity in growth as well as measurement error (following Eaton & Link, 2011) from the capture‐recapture data. I included individuals recaptured up to two times from all available data, with the exception sites with one or fewer recaptures (Balcones, MacDonald Well, Spicewood, Stillhouse, and Tanglewood; Table 1). Several parameters are required to describe a VB growth function: initial size (*s*[*0*]), asymptotic size (*a*), and a growth rate coefficient (*k*). An additional parameter, *λ*, represents individual heterogeneity in the growth curves as the mean to variance ratio (see Eaton & Link, 2011). I estimated parameters *a*,* k*,* λ* as well as standard deviation of measurement error (*SD*). The initial size, *s*[*0*], was fixed at 8 mm based on observations of hatchling growth in captivity to allow for estimation of age‐at‐length. Initial size was estimated using a linear regression of BL vs. age from individual growth of 12 captive hatchlings from 4 to 22 weeks of age (intercept = 8 (*SE* = 0.5), slope = 0.1 (*SE* = 0.007), *r* ^2^ *=* .92). I fit the growth model using MCMC methods in JAGS (Plummer, 2003) and R (R Development Core Team 2016). I ran three chains for 50,000 iterations after a burn‐in of 10,000 and assessed convergence by visually examining trace plots and $\hat{R}$ values. I report posterior means and 95% credence intervals (CRI) for each parameter. I used the mean parameter estimates from the growth curve to estimate the age of initial captures from the VIE data, as some individuals were marked as early as March 2007 from a prior study (O'Donnell & Gluesenkamp, 2009). To estimate longevity*,* I added the time between first and last capture to generate an estimate of age at last capture (following Fellers, Kleeman, Miller, Halstead, & Link, 2013). Additionally, I generated derived estimates from the VB model for the age of mature (gravid) females, body size at 1, 1.5, and 2 years of age, as well as the expected age for a given size based on modes from the size frequency data.

## 3 RESULTS

### 3.1 Size demographics and reproduction

**Figure 3:**
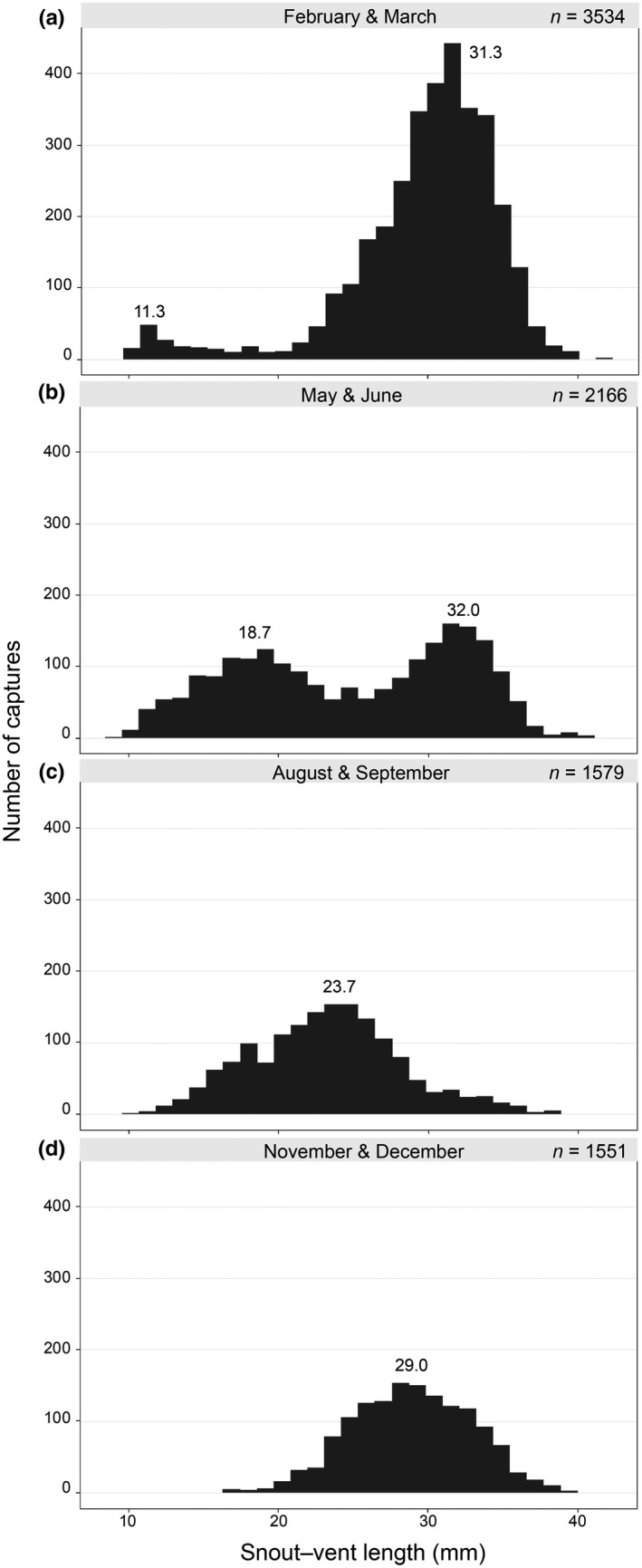
Histogram of snout–vent length (mm) for all *E. tonkawae* captures at 16 sites from 2008 to 2015 during surveys in February and March (a), May and June (b), August and September (c), and November and December (d). Values are shown for the highest density modes

I obtained data from 10,041 captures of 7,315 individual salamanders from 186 surveys at 16 sites, 2008–2015 (Table 1). Seasonal size patterns were relatively consistent among sites and years, so I present the size frequency data as a cumulative summary. Early in the year, the size distribution is mostly unimodal with a heavy left tail representing the youngest size class. Large adults are the dominant size class followed by hatchlings (<12 mm BL; Figure 3a), eventually developing into a strong bimodal pattern from increasing juvenile abundance and recruitment throughout the spring season (Figure 3b). The smaller mode of the size class distribution shifts following growth and recruitment, while the largest individuals (those ≥35 mm SVL) were less abundant during the late summer (Figure 3c). Late fall size distributions were unimodal (Figure 3d) with most individuals representing adults or subadults.

**Figure 4:**
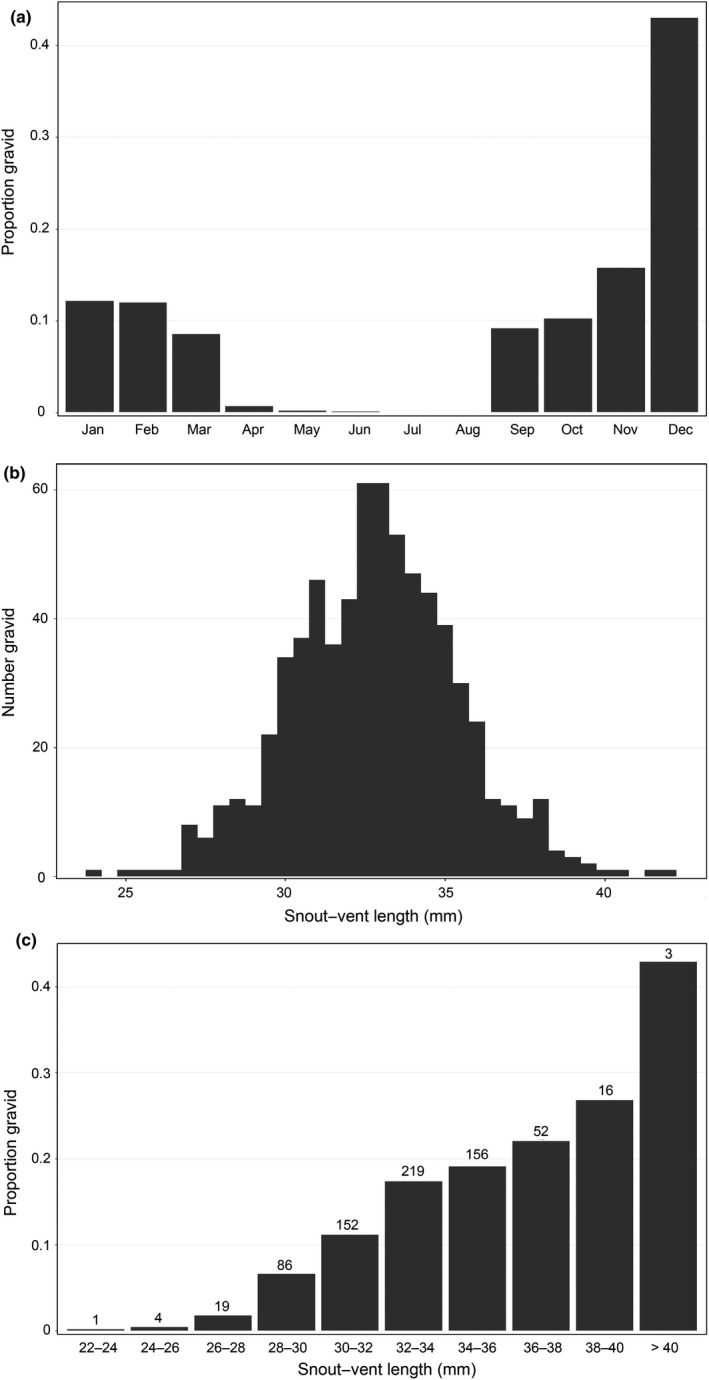
Gravidity in *E. tonkawae* at 16 sites, 2008–2015 (*n* = 708). (a) Proportion of gravid *E. tonkawae* captures by month. (b) Histogram of snout–vent length. (c) Proportion of gravid *E. tonkawae* by size class; total number of gravid individuals observed is indicated above each bar

Overall, gravid individuals accounted for 7.1% of the observations (*n *=* *708). The late fall and winter months included the largest proportions of gravid females (Figure 4a), which peaked in December at 42%. No gravid females were observed during July and August. The minimum gravid size was 23.9 mm SVL (TL = 38.6 mm) and the 5th percentile was 28.4 mm SVL (TL = 48.4 mm) (Figure 4b). The largest individual observed was also a gravid female (SVL = 41.8 mm, TL = 79.5 mm). The proportion of gravid individuals increased as a function of size (Figure 4c). Among recaptured individuals that were gravid on more than one occasion, 35 were recaptured within a 100‐day period. Of these, 74% were gravid during both occasions.

### 3.2 Capture‐recapture models

**Figure 5:**
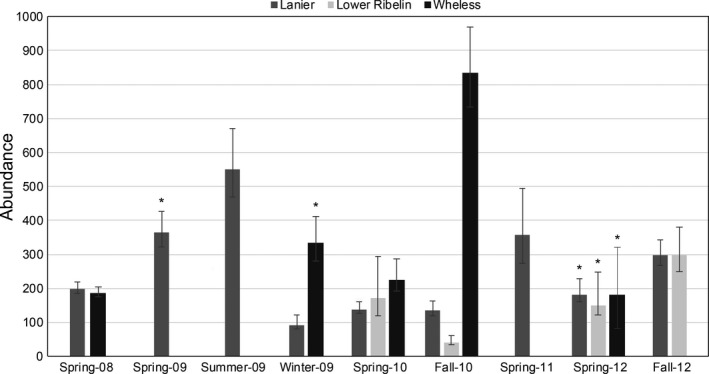
Abundance ($\hat{N}$) of *E. tonkawae* at Lanier, Lower Ribelin, and Wheless springs, 2008–2012. Error bars represent 95% confidence intervals. *indicates estimates following a protracted dry period at the respective site

A severe drought occurred in the region during the first few years of the study, causing several springs to cease flowing. The longest dry period started in 2008 and lasted at least ten months at Lanier and Lower Ribelin, and over a year at Wheless. Shorter dry periods also occurred in 2009 and 2010, followed by another dry period in 2011 at Lanier, Lower Ribelin, and Wheless that lasted several months. Abundances estimated from robust‐design (Lanier) and closed‐population models (Lower Ribelin and Wheless) between 2008 and 2012 were highly variable within and among sites (Figure 5). Wheless had the highest maximum abundance, corresponding to a density of 9.2 individuals/m^2^, followed by Lanier (8.5 ind./m^2^), and Lower Ribelin (5.7 ind./m^2^). Minimum abundance estimates followed the same site pattern of 2.0, 1.4, and 0.78 ind./m^2^, respectively. Estimates of conditional capture probability (p*) ranged from 0.12 to 0.49 at Lanier Spring, from 0.23 to 0.53 at Lower Ribelin, and from 0.17 to 0.34 at Wheless.

Survey intervals ranged from approximately three months to one year at Lanier Spring, precluding the use of any seasonal covariates for modeling movement or survival. I found strong evidence of temporary emigration at Lanier Spring based on the model selection results (Table 2). Temporary emigration followed a Markovian pattern whereby the availability of individuals within the survey area was dependent upon whether they were available or not during the prior survey. Based on higher estimates of ${\hat{\gamma }}^{\prime \prime }$, individuals were more likely to have temporarily emigrated following a summer interval compared to winter (Table 3).

**Table 2:** Model selection results of robust‐design analysis for Lanier Spring, 2008–2012

Model^a^	AICc	ΔAICc	AICc weight	*K*	Deviance	−2log(L)
Markovian emigration: *γ″*(*t*), *γ′*(*t*)	7202.1	0	1	42	8875.9	7116.3
Random emigration: *γ″*(*t*) = *γ′*(*t*)	7222.4	20.3	0	36	8908.7	7149.1
No emigration: *γ″*(*t*) = *γ′*(*t*) = 0	7458.6	256.5	0	29	9159.4	7399.7

ΔAICc = the difference between the AICc score of the current model and top model.

*K *= the number of estimated parameters.

−2log(L) = negative 2 times the log‐likelihood.

a All models had the same model structure for apparent survival, capture and recapture probabilities: *φ*(*t*) *p*(best) *c*(best).

**Table 3:** Temporary emigration and apparent survival estimates for Lanier Spring, 2008–2012, for model *φ*(*t*) *γ″*(*t*) *γ*′(*t*) p*(best) *c*(best)

Period	${\hat{\gamma }}^{\prime \prime }$			${\hat{\gamma }}^{\prime }$			$\hat{\varphi }$		
	Mean	LCL	UCL	Mean	LCL	UCL	Mean	LCL	UCL
Mar‐09	0.49	0.23	0.67	–	–	–	0.73	0.52	1.00
Jun‐09	0.37	0.00	0.54	0.46	0.00	0.79	0.72	0.15	1.00
Dec‐09^s^	0.89	0.78	0.94	0.91	0.00	1.00	0.47	0.20	1.00
Mar‐10^w^	0.41	0.09	0.62	0.86	0.77	0.92	0.48	0.07	1.00
Oct‐10^s^	0.92	0.81	0.97	0.94	0.89	0.97	0.99	0.39	1.00
Apr‐11^w^	0.73	0.46	0.86	0.85	0.72	0.92	1.00	0.44	1.00
Mar‐12	0.00	0.00	0.38	0.22	0.10	0.57	0.11	0.08	0.17

Parameter *γ″*_*k*_ represents the probability an animal in the study area during period *k*−1 moves out of the study area in period *k*;* γ*′_*i*_ is the probability that an animal stays away from the study area in period *k*, given that it was a temporary migration in period *k*−1 (Kendall et al., 1997). *φ*(*t*) = yearly apparent survival probability from time *t*−1 to *t*. Superscripts indicate where the interval (4–7 months) from period *k*−1 to period *k* included either summer (s) or winter (w).

LCL and UCL = lower and upper bounds, respectively, for the 95% confidence interval.

**Figure 6:**
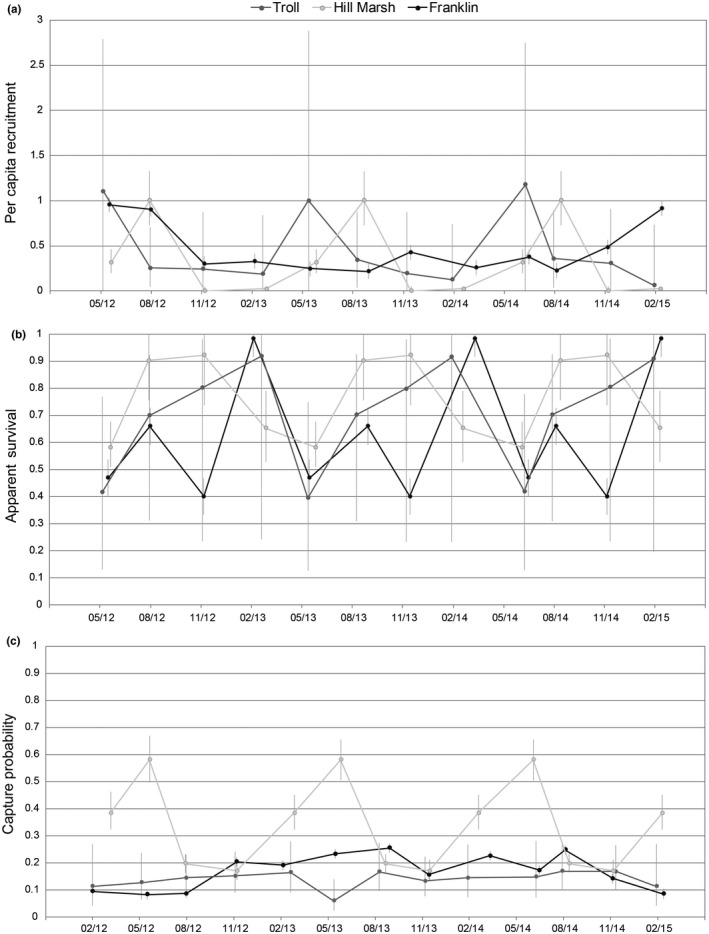
(a) Per capita recruitment ($\hat{f}$) (b) quarterly apparent survival ($\hat{\varphi }$) and (c) capture probability (${\hat{p}}^{0}$) for *E. tonkawae* at three sites, 2012–2015. Error bars represent 95% confidence intervals. Estimates for the last period encompassing multiple seasons are not shown

The best Link‐Barker JS models supported by the data included either season‐ or flow‐dependent survival, per capita recruitment, and capture probability (Table 4). Per capita recruitment was positively correlated with flow rates at Franklin (slope = 0.78, CI = 0.61–0.95), which also exhibited the highest variation in stream flow throughout the study (*SD* = 0.76). Hill Marsh and Troll exhibited much lower variation in stream flow (*SD* = 0.03 and 0.08, respectively); in each case, seasonality was a more important predictor of recruitment, which was highest in the spring or summer and lowest in the fall and winter (Figure 6a). In contrast, models with seasonal variation in apparent survival were best supported for all sites (Table 4). Survival peaked in the winter at Franklin and Troll but was highest in the late summer and fall at Hill Marsh (Figure 6b). Capture probability was negatively correlated with flow at Troll and Franklin, but was seasonal at Hill Marsh (Table 4). Capture probability was generally the lowest at Troll (Figure 6c); this, combined with substantial model selection uncertainty, resulted in high uncertainty of demographic parameter estimates for this site (Figure 6). Hill Marsh had the highest capture probabilities, which varied by season (Figure 6c).

**Table 4:** Model selection results of Jolly–Seber models using the Link‐Barker formulation. *f *= per capita recruitment, the number of individuals at time *t* compared to *t*−1; φ = quarterly apparent survival; *p* ^*0*^ = effective capture probability

Model	AICc	ΔAICc	AICc weight	*K*	Deviance	−2log(L)
Franklin						
*f*(Flow) *φ*(Season) *p* ^*0*^(Flow)	8722.4	0.0	1	8	649.7	8706.3
*f*(Season) *φ*(Season) *p* ^*0*^(Flow)	8749.7	27.3	0	10	673.0	8729.5
*f*(Season) *φ*(Flow) *p* ^*0*^(Flow)	8751.1	28.7	0	8	678.4	8735.0
*f*(Flow) *φ*(Flow) *p* ^*0*^(Flow)	8772.1	49.7	0	6	703.4	8760.0
Hill Marsh						
*f*(Season) *φ*(Season) *p* ^*0*^(Season)	6813.6	0.0	1	14	632.6	6785.3
*f*(Season) *φ*(Flow) *p* ^*0*^ (Season)	6845.6	32.0	0	11	670.8	6823.4
*f*(Season) *φ*(Season) *p* ^*0*^(Flow)	6912.8	99.2	0	12	735.9	6888.5
*f*(Flow) *φ*(Season) *p* ^*0*^(Season)	6917.6	104.0	0	11	742.8	6895.4
Troll						
*f*(Season) *φ*(Season) *p* ^*0*^(Flow)	1480.4	0.0	0.51	12	109.6	1455.2
*f*(Flow) *φ*(Season) *p* ^*0*^(Flow)	1482.0	1.6	0.23	9	117.7	1463.3
*f*(Flow) *φ*(Flow) *p* ^*0*^(Flow)	1483.7	3.3	0.10	6	125.8	1471.4
*f*(Season) *φ*(.) *p* ^*0*^(Season)	1484.5	4.1	0.07	10	118.1	1463.7

ΔAICc = the difference between the AICc score of the current model and top model.

*K *= the number of estimated parameters.

−2log(L) = negative 2 times the log‐likelihood.

Although goodness‐of‐fit tests for robust‐design and Link‐Barker JS models are unavailable, one qualitative way to assess the potential consequences of lack‐of‐fit is to assess the sensitivity of model rankings to changes in $\hat{c}$, a variance inflation factor. Values of $\hat{c}$ from 1.25 to 2.00 did not result in changes to the top capture‐recapture models at Hill Marsh or Franklin. For Troll Spring, models with flow as a covariate were more heavily favored over those with season as $\hat{c}$ increased.

### 3.3 Growth and longevity

**Figure 7:**
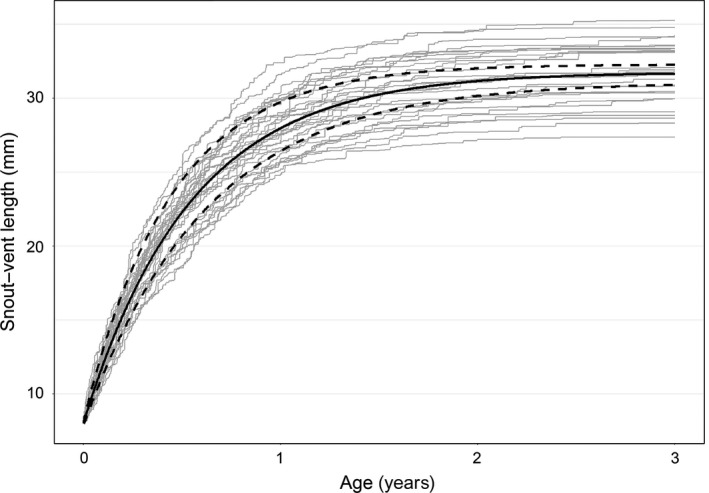
Estimated von Bertalanffy growth curve from hatching (initial SVL = 8 mm) to three years of age for 1,290 *E. tonkawae* recaptured at ten sites, 2008–2015. The dark solid line indicates age‐at‐length estimated from the mean parameter values for *a* and *k* while dashed lines represent age‐at‐length estimated from the upper and lower 95% credence intervals for *a* and *k*, respectively. Light gray lines are from 30 randomly generated growth curves from the mean parameter estimates to demonstrate individual variation

The VB growth model revealed high variation of individual growth trajectories for 1,290 salamanders among ten sites (*λ* = 5.56, [4.06–7.57]; Figure 7). Asymptotic size was reached in approximately 2 years (assuming initial size = 8 mm SVL) at 31.73 mm (CRI = 31.21–32.28) with a growth rate coefficient of 5.52 (CRI = 4.76–6.34). The asymptotic size corresponds to the 72nd percentile of SVL among all salamanders measured. The largest individual was a gravid female over 30% greater than the estimated asymptotic size. The *SD* of measurement error was 0.92 mm (CRI = 0.79–1.06).

The expected size (mm SVL) of a typical individual from the data at 1, 1.5, and 2 years of age was 28.53 (CRI = 27.61–29.20), 30.55 (CRI = 30.10–30.96), and 31.29 (CRI = 30.88–31.70), respectively. Based on the 5th percentile gravid size, age at maturity was 11.8 months (CRI = 10.6–13.3). Using only the growth model, age estimates are not possible beyond the asymptotic size. I used the growth curve to estimate age at initial capture for all 1,418 VIE recaptures then added the time between first and last capture to generate an estimate of age. I assumed individuals at or greater than the asymptotic size at initial capture were at least two years old. For recaptured adults (≥1 year old), the median age was 2.3 years, the 75th percentile was 4.0 years, and the maximum was 7.9 years.

The approximate age for young‐of‐year from the size distribution data was 0.9 months (CRI = 0.79–1.02) for the 11.3 mm mode in February/March (Figure 3a). This was followed by a larger 18.7 mm peak in May/June at 3.6 months (CRI = 3.2–4.1), which corresponds to an average age difference of 2.7 months between the first two quarters. This is roughly equivalent to the average survey interval (approximately three months). Similarly, growth from 18.7 mm to the following mode of 23.7 mm (mean age = 6.5, CRI = 5.7–7.3) in August/September corresponds to a 2.9‐month age difference.

## 4 DISCUSSION

Population demographics of *E. tonkawae* follow a predominantly seasonal pattern. Gravid females first appeared in September and were most abundant during the late fall and winter. The proportion of gravid individuals observed also increased as a function of body size. Larger females may be more abundant than large males (due to sex‐biased survival or growth) or may be gravid more frequently than smaller females (higher fecundity). For example, numerous individuals were gravid more than once during a season, indicating *E. tonkawae* may lay multiple clutches per year. Oviposition likely occurs below the surface throughout the fall and winter, as I observed only a single egg during this study. Peak gravidity in December followed by peak hatchling (<12 mm SVL) abundance in February is consistent with the timing of egg incubation (1 month) and yolk sac absorption (2 weeks) in the closely related *E. sosorum* (Cantu, Crow, & Ostrand, 2016).

While seasonal reproduction is common among *Eurycea* and many other salamanders in temperate regions (Bruce, 2005; Petranka, 1998), reproductive phenology is not well documented for most central Texas *Eurycea*. Seasonality in reproduction was suggested for *E. tonkawae* (Bowles et al., 2006), *E. neotenes* (Bogart, 1967), and transforming populations of *E. troglodytes* (Sweet, 1977), but is well documented for *E. naufragia* (Pierce et al., 2014). These species occupy smaller springs compared to those inhabited by *E. nana* and *E. sosorum*. *Eurycea nana* is restricted to one of the largest springs in Texas (San Marcos Springs) and reproduces year‐round (Nelson, 1993; Tupa & Davis, 1976). Based on juvenile (individuals <25 mm TL) abundance data, reproduction in *E. sosorum* (at Barton Springs) also appears to be nonseasonal (Dries, 2012; Gillespie, 2011). Differences in reproductive phenology and recruitment may be a function of spring size and reliability. For example, per capita recruitment of *E. tonkawae* varied seasonally at Hill Marsh, which is an isolated, low‐discharge spring. This contrasts with Franklin, where spring and stream flow from its large watershed was a better predictor of recruitment compared to seasonality. Large spring systems may be less susceptible to variation in environmental conditions caused by terrestrial seasonality compared to smaller, intermittent springs and stream habitats.

The VB model indicated substantial variation in growth of *E. tonkawae*. This is partly a consequence of combining data from multiple populations and across a range of environmental conditions, although it is possible to directly account for environmental stochasticity in the hierarchical VB model (e.g., Connette, Crawford, & Peterman, 2015). Nevertheless, the VB model used here is useful for approximating average age‐at‐length with two primary assumptions: (1) variance in initial size is negligible; (2) generalizations from the data are applicable to the species at large. Based on the minimum estimated age at maturity for females as well as time required for oviposition and incubation in similar species (Cantu et al., 2016), the generation time for *E. tonkawae* is probably between 1 and 1.5 years. Female size at maturity (ca. 24–28 mm) was within the range of that reported for *E. troglodytes* (25 mm SVL; Bruce, 1976; Sweet, 1977; sensu Chippindale et al., 2000) and *E. naufragia* (25.9 mm head–trunk length; Pierce et al., 2014), but larger than *E. nana* (21 mm SVL; Tupa & Davis, 1976).

While some individuals were estimated to be eight years of age when last captured, this is likely a conservative estimate of longevity. Determination of longevity is difficult, particularly given that the oldest individuals in a population are encountered with low frequency. In this case, the VB asymptotic size estimate does not represent the maximum size achieved, but the mean maximum size (e.g., see Staub (2016) for a discussion of longevity estimation using VB models), and many individuals in the population were larger. For example, 59 *E. tonkawae* were at or above the mean asymptotic size when initially captured and were later recaptured up to six years later. One very large individual (42 mm SVL at time of initial capture, 30% greater than the VB asymptotic size) from a cave population had been repeatedly captured over a 14‐year period (A. Gluesenkamp and M. Sanders, personal communication), although individuals in caves may live longer than their surface counterparts. Longevity estimates of wild plethodontid salamanders range from 7 to 36 years (Staub, 2016) although estimates have not been reported previously for central Texas *Eurycea* species. Several longevity records exist for captive populations (e.g., Herald, 1955) including the City of Austin's captive refugium, where several *E. tonkawae* are over 11 years of age, while the oldest *E. sosorum* is 17.5 years old (D. Chamberlain, personal communication; City of Austin, unpublished data). Thus, longevity may extend well beyond eight years in *E. tonkawae*.

Changes in size demographics from season to season were consistent with predicted growth rates, illustrating a pattern of recruitment for a single age‐class of first‐year salamanders. Per capita recruitment estimates were somewhat consistent with this as well, which indicated that most individuals at Hill Marsh and Troll springs (≥16 mm SVL) entered the population during the spring and summer (although recruitment was flow‐dependent at Franklin). On average, young‐of‐year hatch in the winter and females reach maturity as early as the late summer/fall. Interestingly, most individuals observed in the late summer (August and September) were recently recruited first‐year adults or smaller, with a relatively low ratio of second‐year and older individuals (i.e., those ≥31.29 mm based on the VB model). Given that most recaptured individuals were in the latter age class, this raises the question: Why were so few large adults typically observed in that season? Bruce (1976) found a similar pattern in *E. troglodytes*, but suggested low recruitment rates (i.e., low survival of juveniles) to explain this. Seasonal migration might also explain the apparent disparity in size‐ and age‐class frequencies during the late summer. The probability that individuals temporarily emigrated following summer intervals (given they were present during the prior survey) tended to be higher than for periods that follow winter intervals at Lanier Spring. Additionally, capture probability was lowest during the late summer and fall at Hill Marsh. These examples may reflect movement patterns of the second‐year and older individuals, although this is speculative because I did not explicitly test for either a size or season effect of temporary emigration due to limitations of the data. Provided the phenomenon of seasonal migration is real, its biological significance is unclear. Large salamanders may retreat underground in response to (or anticipation of) lower flows during the driest part of the year, or perhaps move to avoid competition with recently recruited first‐year adults.

Temporary emigration can result in biased parameter estimates of the JS model when the pattern of emigration is Markovian (Kendall et al., 1997). In one study, JS estimates of population size were found to be generally unbiased when animals had a high probability of returning to the study area, but were negatively biased when they had a low probability of returning (Zehfuss, Hightower, & Pollock, 1999). Because recruitment is a function of population size, the direction of bias may be similar. At Lanier Spring, temporary emigration was Markovian, although estimates from a prior single‐year study with shorter, monthly sampling intervals at Lanier, Lower Ribelin, and Wheless indicated random emigration (O'Donnell & Gluesenkamp, 2009). In the case of random emigration, JS capture probability is the product of the probability of capture and the probability of temporary emigration, while the other parameters are unbiased (Kendall et al., 1997; Nichols, 2016). Thus, it is unclear the degree to which per capita recruitment estimates may be affected in this study because the form (Markovian or random), and the magnitude of temporary emigration is unknown. Another consideration in the interpretation of recruitment from the JS models is that it includes both births (in this case, entrants of small juveniles) as well as immigration (e.g., the return of temporary migrants). Despite these challenges, it is encouraging that timing and pattern of recruitment predicted by the JS models was generally consistent with the size frequency distribution, gravidity, and growth data.

For many sites it was difficult to obtain open‐population JS estimates due to low recapture probabilities. This was partly a consequence of decisions regarding the study design, such as survey interval (quarterly at most sites) and the area surveyed (limited to areas near spring outlets). However, the tendency for salamanders to migrate from the vicinity of springs (this study, Bendik et al., 2016), their ability to retreat to subterranean habitats (Bendik & Gluesenkamp, 2013), and periodic drying of surface habitats likely influenced recapture rates as well. Federal guidelines for surveys of *E. tonkawae* and two other federally listed congeners (*E. chisholmensis* and *E. naufragia*) recommend that researchers use an open‐population survey design for estimating demographic parameters (US Fish and Wildlife Service 2014b). However, based on the results of this study, I advise using closed and or robust‐design methods in lieu of an open‐population design for long‐term investigations of population demographics of *E. tonkawae*, particularly when survey intervals are 3 months or greater.

In general, abundance estimates were consistent with those reported previously at the same sites during a single‐year study (O'Donnell & Gluesenkamp, 2009), but within‐site variability was greater. Large shifts in abundance occurred, even within a single year. For example, the lowest abundance at Lanier during the 4‐year period was observed in December 2009 ($\hat{N}$ = 91), despite the highest abundance occurring just 6 months earlier following a protracted dry period ($\hat{N}$ = 551). In comparison, $\hat{N}$ ranged from approximately 80 to 250 during eight surveys in 2007 at Lanier (figure 5 in O'Donnell & Gluesenkamp, 2009). Dramatic changes in abundance are not uncommon for amphibian populations, particularly in response to environmental stochasticity (Semlitsch, Scott, Pechmann, & Gibbons, 1996). These temporal dynamics are consistent with the high temporal variation in counts of *E. tonkawae* (Bendik et al., 2014; Bowles et al., 2006). This may be driven by variation in reproductive output, migration patterns, or mortality due to adverse environmental conditions. However, abundance following long dry periods was not disproportionate to estimates from other periods as might be expected given the negative impacts of drought on body condition of *E. tonkawae* (Bendik & Gluesenkamp, 2013). Remarkably, some recruitment still occurred during drought, as several juveniles were observed only a few weeks following a ten month period of completely dry surface conditions (March 2009, Lanier Spring; Bendik & Gluesenkamp, 2013). It is worth noting, however, on several occasions I observed stranded juvenile salamanders under rocks immediately following the cessation of spring flow; a similar observation was made by Sweet (1977). Thus, the apparent population structure may be determined in part by spring flow (this study; Sweet, 1977) and a size bias in the ability of individuals to migrate into subterranean habitats as springs cease flowing.

Collectively, the results presented here suggest that life history characteristics of *E. tonkawae* help facilitate a resiliency to drought and an ability to respond quickly to favorable environmental conditions (e.g., via short generation times and multiple clutching). Adaptations to temporally variable flow are likely common among epigean central Texas *Eurycea* populations, as spring reliability varies widely throughout the Edwards Plateau (Sweet, 1982). Although urbanization remains the primary near‐term threat to many populations of *E. tonkawae* (Bendik et al., 2014), adaptations that allow them to cope with variable flow conditions may become more important as a drier future looms over central Texas aquifers (Stamm et al., 2015).

## CONFLICT OF INTEREST

None declared.
